# 3D Visible Light-Based Indoor Positioning System Using Two-Stage Neural Network (TSNN) and Received Intensity Selective Enhancement (RISE) to Alleviate Light Non-Overlap Zones

**DOI:** 10.3390/s22228817

**Published:** 2022-11-15

**Authors:** Li-Sheng Hsu, Chi-Wai Chow, Yang Liu, Chien-Hung Yeh

**Affiliations:** 1 Department of Photonics & Graduate Institute of Electro-Optical Engineering, College of Electrical and Computer Engineering, National Yang Ming Chiao Tung University, Hsinchu 30010, Taiwan; 2 Department of Photonics & Graduate Institute of Electro-Optical Engineering, College of Electrical and Computer Engineering, National Chiao Tung University, Hsinchu 30010, Taiwan; 3 Philips Electronics Ltd., N.T., Hong Kong; 4 Department of Photonics, Feng Chia University, Taichung 40724, Taiwan

**Keywords:** optical wireless communication (OWC), visible light communication (VLC), visible light positioning (VLP), light emitting diode (LED), machine learning

## Abstract

The high precision three-dimensional (3D) visible light-based indoor positioning (VLIP) systems have gained much attention recently for people or robot navigation, access tracking, etc. In this work, we put forward and present the first demonstration, up to the authors’ knowledge, of a 3D VLIP system utilizing a two-stage neural network (TSNN) model. The positioning performance would degrade when the distance between the light emitting diode (LED) plane and the receiver (Rx) plane increases; however, because of the finite LED field-of-view (FOV), light non-overlap zones are created. These light non-overlap zones will produce high positioning error particularly for the 3D VLIP systems. Here, we also propose and demonstrate the Received-Intensity-Selective-Enhancement scheme, known as RISE, to alleviate the light non-overlap zones in the VLIP system. In a practical test-room with dimensions of 200 × 150 × 300 cm^3^, the experimental results show that the mean errors in the training and testing data sets are reduced by 54.1% and 27.9% when using the TSNN model with RISE in the *z*-direction, and they are reduced by 39.1% and 37.8% in the *xy*-direction, respectively, when comparing that with using a one stage NN model only. At the cumulative distribution function (CDF) P90, the TSNN model with RISE can reduce the errors by 36.78% when compared with that in the one stage NN model.

## 1. Introduction

Due to the recent development of Internet-of-Things (IOT), augmented reality/virtual reality/mixed reality (AR/VR/MR), autonomous mobile robot (AMR) and unmanned aerial vehicle (UAV) services, wireless sensor networks, etc., high precision positioning is highly desirable. In some scenarios, high precision positioning is critically important. For example, in factories where people and robots share the same working environment, precise positioning of workers and robots is crucial for performance improvement and safety issues. Moreover, in the hospitals where medical equipment can be efficiently tracked and located could be a matter of life or death. The popular Global Positioning System (GPS) needs to obtain positioning data from satellites; hence, it may not provide high enough accuracy for the positioning in the indoor environments. Recently, visible light communication (VLC) and light fidelity (Li-Fi) have been proposed [1,2,3,4,5,6,7], and it can provide the value-added functions of lighting, communication and indoor positioning, known as visible light indoor positioning (VLIP) using the already installed light-emitting-diode (LED) lighting networks. VLC is also considered one of the promising candidates for the 6G mobile and wireless systems [8]. In the literature, different VLIP schemes were proposed and demonstrated. One scheme is based on proximity [9]. It was realized by identifying the received optical identifier (ID) and lookup table for the positioning, and its accuracy depended on the illumination zone of each LED. Another scheme is based on time-of-arrival (TOA)/time-difference-of-arrival (TDOA) [10,11]. It was realized by analyzing the time or differential time of the received optical signal for positioning. However, it needed precise synchronization between the transmitters (Txs) and receivers (Rxs), and could complicate the positioning system. Moreover, angle-of-arrival (AOA) scheme is also one the candidates for VLIP [12,13]. It was realized by analyzing the photocurrents of different location photodiodes (PDs) for the positioning. In this scheme, angular diversified Rx was needed, and the footprint of the Rx could be large. Moreover, asynchronous VLIP using orthogonal pseudo-random codes [14] is also another candidate. It was realized by using different orthogonal codes; however, it may need large computation and will rise the latency.

Among these VLIP systems, received-signal-strength (RSS) VLIP systems have gained much attention recently [15,16]. Since the received optical intensity decreases when the distance between the Rx and Tx increases, the VLIP can be realized by analyzing the RSS or the received optical power from several LEDs (i.e., the channel information), and then the positioning data can be easily estimated by using trilateration. However, the received optical powers from different LEDs may not be so easy to acquire in practice. A large number of RSS location data should be acquired to build a lookup table for accuracy positioning. Additionally, the emitted optical powers from LEDs may change due to the aging issues. To solve the issue of the requirement of a large number of RSS data, interpolation to increase the number of RSS data [17], as well as positioning unit cell copying using transfer learning [18] were reported. Moreover, in order to reduce the positioning error, machine learning and deep learning (ML/DL) technologies were utilized. Table 1 illustrates the recent 3-dimensional (3D) RSS-based VLIP systems. In 2017, Guan et al. demonstrated a 3D RSS VLIP scheme realized by using code division multiple access (CDMA) [19]. In 2018, Zhou et al. reported that a 3D VLIP can also be realized by a particle-assisted stochastic search (PASS) algorithm for mitigating the non-convex optimization issue of the nonlinear RSS VLC model [20]. In 2019, Plets et al. illustrated a 3D RSS VLIP scheme using trilateration together with a nonlinear least squares (NLLS) using simulations [21] and later, using experiment [22]. RSS VLIP utilizing neural networks to enhance the positioning accuracy is also very popular. In 2019, Zhang et al. illustrated by simulation a 3D indoor VLIP system based on artificial neural network (ANN) and hybrid RSS/phase-differences-of-arrival (PDOA) [23]. Also in the same year, Du et al.’s experiment demonstrated a 3D VLIP using RSS trilateration with DL technique [24]. Additionally, He et al. also experimentally illustrated a 3D VLIP using ANN [25]. Although a high accuracy was obtained, the unit cell is small and may not be applicable in practical scenarios. In 2020, Wu et al. reported a 3D VLIP system. In order to reduce the computation complexity, instead of using ANN, kernel ridge regression (KRR) was utilized [26].

In this work, we put forward and illustrate the first demonstration, up to our knowledge, of a 3D VLIP system utilizing RSS and two-stage neural network (TSNN) model. The positioning performance would degrade when the distance between the LED plane and the Rx plane increases; however, because of the finite LED field-of-view (FOV), light non-overlap zones are created. These light non-overlap zones will produce high positioning error particularly for the 3D VLIP systems. Here, we also propose and demonstrate a Received-Intensity-Selective-Enhancement scheme, known as RISE, to alleviate the light non-overlap zones in the VLIP system. In a practical test-room with dimensions of 200 × 150 × 300 cm^3^, the experimental results show that the mean errors in the training data set, testing data set, and testing data standard deviation are reduced by 54.1%, 27.9%, and 0.2% when using the TSNN model with RISE in the *z*-direction, and are reduced by 39.1%, 37.8%, and 35.9% in the *xy*-direction, respectively, when compared with that using the one stage NN model only.

## 2. TSNN 3D VLIP Experiment and Algorithms

### 2.1. Experimental Setup

**Figure 1:**
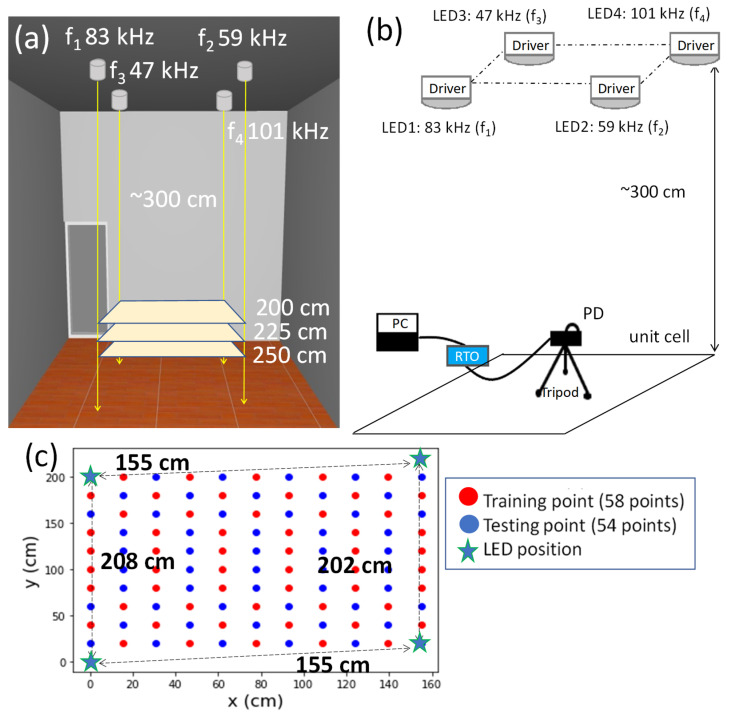
(**a**) Architecture and (**b**) experimental setup of the proposed 3D RSS VLIP system. (**c**) Top-view of the VLIP layer, illustrating the LED lamps, testing, and training locations.

Figure 1a,b present the architecture of the experimental setup of the proposed 3D RSS VLIP system. The distance between the ceiling and the floor is ~300 cm. The positioning area has four LED lamps (TOA^®^ LDL030C) with 13 W output power each. Moreover, each LED is encoded by a 3.125 kbit/s Manchester-coded (MC) ID, which is frequency upconverted to specific RF carrier frequency. The RF upconverted signals are used to modulate the LED lamps via bias-tee circuits. The PD (Thorlabs^®^ PDA36A) is attached to a real-time-oscilloscope (RTO, PicoTechnology^®^ ps5432d) for RSS optical signal recording. The PD has the detection wavelength window of 350–1100 nm, and the optical-to-electrical bandwidth from DC—10 MHz. The carrier frequencies are between the bias-tee cut-off frequency (i.e., ~10 kHz) and the LED lamp bandwidth (i.e., ~1 MHz). Hence, we select 47 kHz, 59 kHz, 83 kHz, and 101 kHz. The odd frequencies are used to alleviate overlapping of the harmonic frequencies after the signal detection. We store the training data and testing data from 3 layers at different heights, and the distance is 250, 225, and 200 cm from the LED plane. Figure 1b shows the top-view of the VLIP layer, illustrating the training, testing, and LED locations. In this practical experimental test-bed, the unit cell is not a perfect rectangle, and the size is about 155 cm × 200 cm. For each layer, we measure 112 location points, of which 58 location points are for training and 54 location points are for testing. Each location point is measured by 20 times. Therefore, the training set has a total of 3480 data (58 locations × 20 measurements × 3 layers), and the testing set has 3240 data (54 locations × 20 measurements × 3 layers).

**Figure 2:**
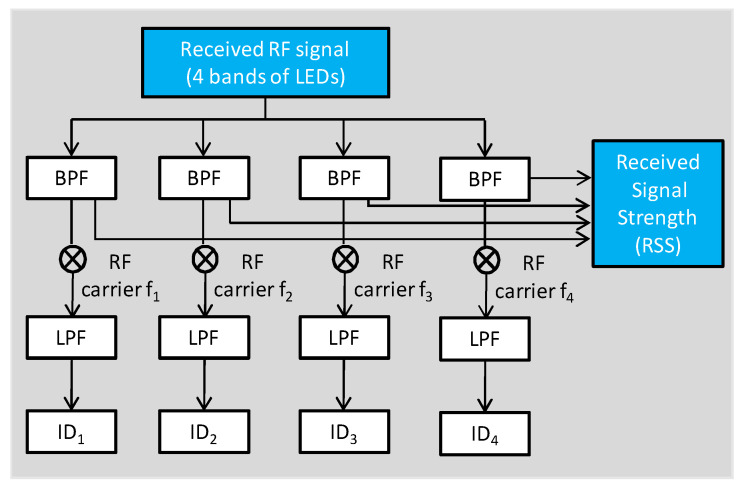
Rx architecture to decode the 4 IDs and 4 RSS values. BPF: band-pass filter; LPF: low-pass filter.

Figure 2 shows the Rx architecture. A single PD receives the signal bands from four LEDs simultaneously for the positioning. It is worthwhile to note that there is no need for the PD to receive optical signals from the 4 LEDs at the same time, since the machine learning model is built from the actual indoor environmental training data, and the RSS features extracted from the training data have already taken into account that some locations may not have all the 4 RSS values (*p*_1_, *p*_2_, *p*_3_, *p*_4_) simultaneously. There is a wall near one side of the unit cell, and the distance between the wall and the unit cell is ~2 m. We do not notice any reflection for the wall. The RTO carries out the analog-to-digital conversion (ADC). Then the optical MC IDs and RSS signals are retrieved as shown in Figure 2. The four optical IDs can determine which unit cell the Rx is located, and the four RSS values can predict the coordinates of the Rx inside that unit cell. As shown in Figure 2, band-pass filters (BPFs) at specific frequencies of 47 kHz, 59 kHz, 83 kHz, and 101 kHz retrieve the RSS values. Afterward, each signal band is also down-converted, and the optical ID is retrieved by a low-pass filter (LPF). During the NN training, at each physical location, the 4 RSS values (*p*_1_, *p*_2_, *p*_3_, *p*_4_) are used as the features of the VLIP model, and the corresponding coordinates (*x*, *y*, *z*) are used as the labels of the model. Moreover, in order to increase the positioning accuracy, the total RF signal strength at each location is also used as one of the features for the VLIP model.

**Figure 3:**
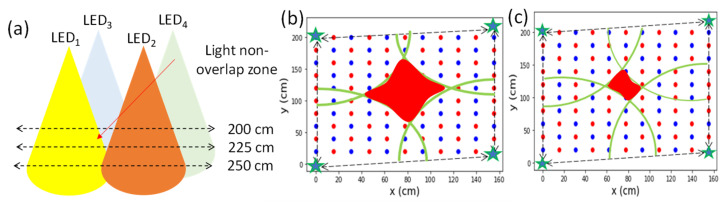
(**a**) Illustration of light non-overlap zone produced by the 4 LEDs. Top views of light non-overlap zone at Rx plane (**b**) 200 cm and (**c**) 225 cm.

It is generally consider that the performance of VLIP systems would decrease if the separation between the LED and the Rx increases due to the reduction of the optical signal-to-noise ratio (SNR). However, owing to the finite LED lamp FOV, light non-overlap zones are created. These light non-overlap zones could be large and could introduce high positioning error since no light can be detected at these zones as shown in Figure 3a. Figure 3b,c are the top views of the illuminated areas at the 200 and 225 cm Rx planes away from the LED plane, respectively. We can observe that the light non-overlap zone (red region) is bigger at the 200 cm plane, and the illuminated zones (green line regions) of the four LEDs are smaller.

After collecting the 4 RSS data and total RF signal strength, we will perform the data pre-processing. There are two parts in the data pre-processing: the first part is the Z-score normalization process, and the second part is the feature expansion. First, the 4 RSS values are Z-score normalized based on Equation (1),

(1) $$z(p_{i})=\frac{p_{i}-\mu_{i}}{\sigma_{i}}$$

where *p_i_* is to the RSS value of the *i*-th LED, and *σ_i_* and *μ_i_* are the standard deviation and mean of the *i*-th LED lamp. Z-score normalization is performed to rescale the measured RSS values at different Rx planes to have similar intensity ranges. After Z-score normalization, the RSS values of LED at Rx planes of 200 cm, 225 cm, and 250 cm can be rescaled within similar intensity range. This process can speed up the convergence during the machine learning model training.

The second process is the feature expansion. This process is to increase the number of input features for the machine learning model, so that the accuracy of the model prediction can be increased. In the feature expansion process, the original 4 RSS feature values (*p*_1_, *p*_2_, *p*_3_, *p*_4_) are multiplied with itself and among themselves to generate 14 RSS feature values. The 14 RSS feature values include the original first-order terms, their second-order terms, and the cross-multiplication terms, as shown in Equation (2).

(2) $$RSS_{feature}=[p_{1},p_{2},p_{3},p_{4},\cdots ,p_{1}p_{2},p_{1}p_{3},p_{1}p_{4},\cdots ,p_{1}^{2},p_{2}^{2},p_{3}^{2},p_{4}^{2}]$$

After the data pre-processing process, the training set is applied to the NN model. In this paper, we will compare three cases: the one stage NN model, the TSNN model, and the TSNN model with the RISE.

### 2.2. One Stage NN Model

**Figure 4:**
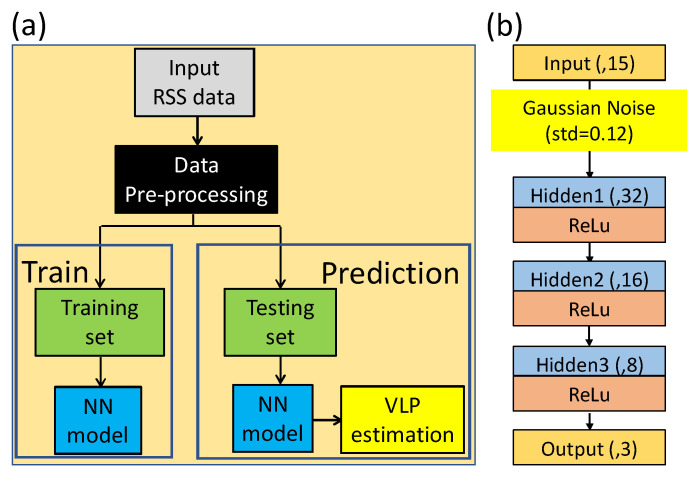
(**a**) Flow chart of the one stage NN model. (**b**) The structure of the one stage NN model.

Figure 4a shows the flow chart of the one stage NN model for the 3D VLIP system. First, the 4 RSS data and the total RF signal strength are used as the input data (gray block). Then, data pre-processing which was mentioned before is proceeded. Then the data are divided into training set and testing set based on their locations depicted in Figure 1c. After the training process, the NN model is built. Then, the testing set is applied to estimate the 3D VLIP coordinates. Figure 4b illustrates the architecture of the NN model. It consists of 5 layers, including 1 input, 1 output, and 3 hidden layers. As illustrated in Figure 4b, there are 15 nodes at the input layer (i.e., labeled as input(,15)) representing the 14 RSS feature values and 1 total RF signal strength. The nodes of 3 hidden layers are 32, 16 and 8, respectively, and they are also fully connected (FC). Moreover, the output of each hidden layer will go through the activation function of a Rectified Linear Unit (ReLU). There are 3 nodes at the output layer representing the predicted *x*, *y*, and *z* coordinates of the Rx. We put a Gaussian noise layer (standard deviation = 0.12) between the input layer and first hidden layer for data augmentation. The optimizer and loss function are Adam and mean square error (MSE), respectively. The training epochs are 300.

### 2.3. Two Stage NN (TSNN) Model

**Figure 5:**
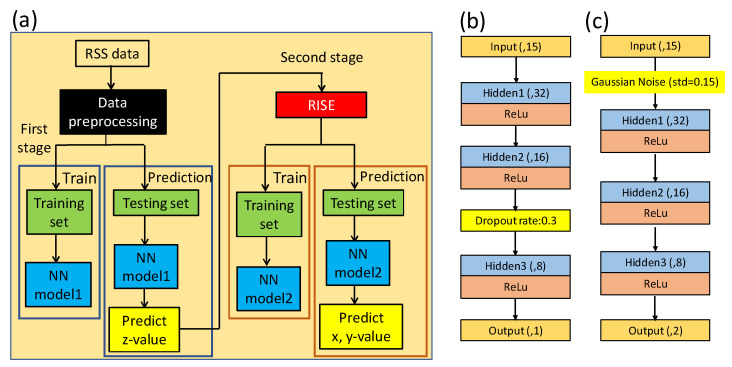
(**a**) Flow chart of the two stage NN (TSNN) model. (**b**,**c**) Structures of NN models.

Figure 5a shows the flow diagram of TSNN model for the 3D VLIP system. The first stage of the TSNN is almost the same as the one stage NN model and the difference is on the structure of the NN model as illustrated in Figure 5b. The nodes of output layer in the first stage of the TSNN is changed to 1 representing the *z* coordinate, and we use a dropout layer (rate = 0.3) instead of Gaussian noise layer to avoid over-fitting. The optimizer and loss function are the same, but the training epoch is 400. After the prediction of the *z* coordinate, the proposed RISE (i.e., red block in Figure 5a) is executed. The details of the RISE will be discussed later in this section. After the RSS data enhancement, the data will be proceeded by the second stage of the TSNN. The structure of the second stage of the TSNN model is illustrated in Figure 5c. The nodes of input layer are still 15, which consist of the 14 terms from the second-order transformation and the predicted z coordinate from the first stage of TSNN. The standard deviation in Gaussian noise layer is increased to 0.15, and the nodes of output layers are 2 which represent the predicted *x* and *y* coordinates. The remaining parameters of the second stage TSNN model are the same as the first stage TSNN.

**Figure 6:**
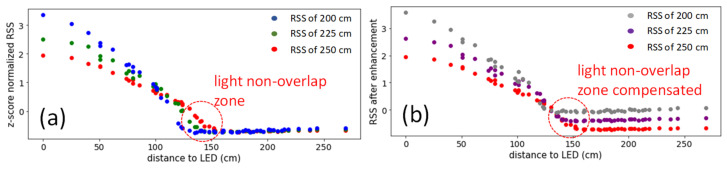
(**a**) LED_1_ light distribution profile in each Rx plane. (**b**) The LED1 light distribution profile after RISE.

As we mentioned before, at the 225 and 200 cm Rx planes, there are light non-overlap zones. Figure 6a illustrates LED light distribution profile in different Rx planes. Without the loss of generality, we use LED_1_ as an example. The x-axis of Figure 6a,b is the distance between the measuring point and LED lamp; 0 cm represents the location directly under the LED_1_. We can see that at the region around 150 cm (red dotted circle), when the distance between the LED and Rx plane decreases, for example, at the 200 cm Rx plane, RSS values drop rapidly. This is the light non-overlap zone, and the *x* and *y* locations should be consistent at different LED planes.

Here, we discuss the proposed RISE (i.e., red block in Figure 5a). In the TSNN, the vertical z-value should be firstly predicted. Once the vertical z-value is obtained, we can calculate the height of the Rx with respect to the reference (or standard) Rx plane. In this experiment, the reference Rx plane is set at the 250 cm Rx plane since there is no light non-overlap zone at this Rx plane. The RISE process is to compensate the light non-overlap zones based on the ratio of the other illuminated region. The RISE process is as shown in Equation (3),

(3) $$RSS_{enhanced}=RSS_{original}+\frac{(h_{std}-h_{t\arg et})\tan\theta }{h_{std}\tan\theta }\alpha$$

where *h_std_* is the height of the standard (or reference) layer, which is set at the 250 cm Rx plane as discussed before since there is no light non-overlap zone, and *h_target_* is the height of the target layer (i.e., 225 or 200 cm). *θ* is the divergence angle of the LED lamp, which is set at 32 degrees based on our measurement results. By multiplying the tan*θ* and the height, we can obtain the size of the illuminated region of the LED lamp at this height which is the denominator. For the numerator, we can calculate the extent to which the LED illuminated region is reduced due to the height. After knowing the reduced ratio at different heights, we will multiply an *α* to control the compensation level of the RSS value. Figure 6b reveals the RSS distribution after the RISE, illustrating that the light non-overlap zone, particularly around 150 cm, can be compensated. When comparing Figure 6a,b, it can be observed that in Figure 6b, the RSS value at the vertical 200 cm plane is higher than that at the 225 cm plane, and the 250 cm plane around the 150 cm horizontal direction. This illustrates that the light non-overlap zone has been compensated.

## 3. Results and Discussion

**Figure 7:**
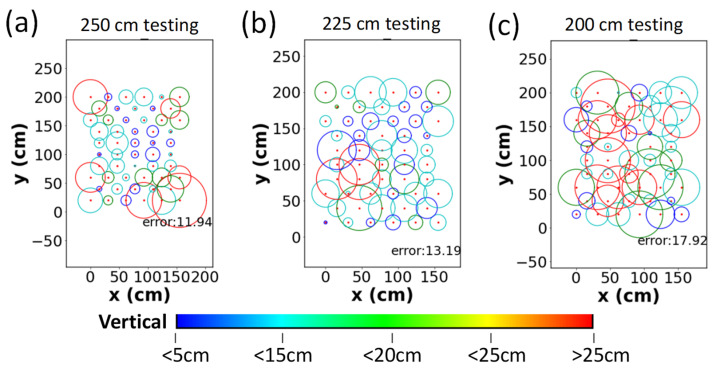
Experimental average error distributions of the testing points using the one stage NN at the (**a**) 250 cm, (**b**) 225 cm, (**c**) 200 cm Rx planes.

When one stage NN model is employed, the mean and standard deviation of errors for the testing data set in the vertical direction (*z*-direction) are 11.39 and 9.10 cm, respectively. The mean and standard deviation of errors and for the horizontal direction (*xy*-direction) are 12.79 and 7.99 cm, respectively. Figure 7a–c show the average error distributions of the testing data set at different Rx planes. The red dots are the location of the testing points. The radius and color of circle represent the average error in the *xy*- and *z*-directions, respectively. When the height increases, the average error in the *xy*-direction becomes larger. The average errors are 11.94, 13.19, and 17.92 cm at the 250, 225, and 200 cm Rx planes, respectively. Moreover, the average error in the *z*-direction is also large at the 200 cm Rx plane as illustrated by many red circles in Figure 7c.

**Figure 8:**
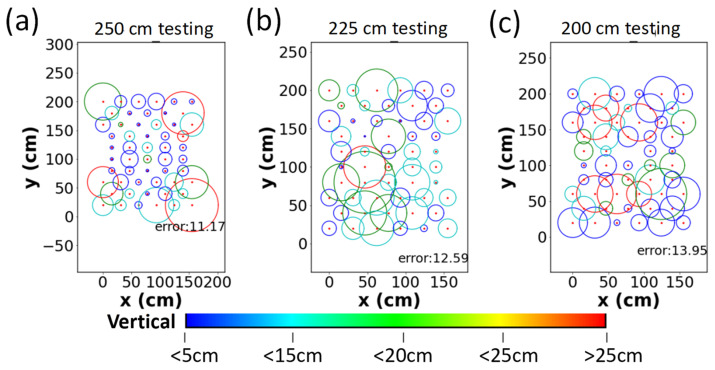
Experimental average error distributions of the testing points using the TSNN without RISE at the (**a**) 250 cm, (**b**) 225 cm, (**c**) 200 cm Rx planes.

When the TSNN model without the RISE is employed, the mean and standard deviation of errors for the testing data set in the vertical direction (*z*-direction) are 8.80 and 9.90 cm, respectively. The mean and standard deviation of errors and for the horizontal direction (*xy*-direction) are 12.56 and 8.30 cm, respectively. Figure 8a–c show the average error distributions of the testing data set at different Rx planes. We can observe that when using the z coordinate from the first stage of the TSNN as the input feature to the second stage of the TSNN, the positioning error can be reduced; however, the standard deviation of the error is still large. Figure 8a–c also illustrate the average error at each Rx planes, and the average errors are 11.17, 12.59, and 13.95 cm at the 250, 225, and 200 cm Rx planes, respectively.

**Figure 9:**
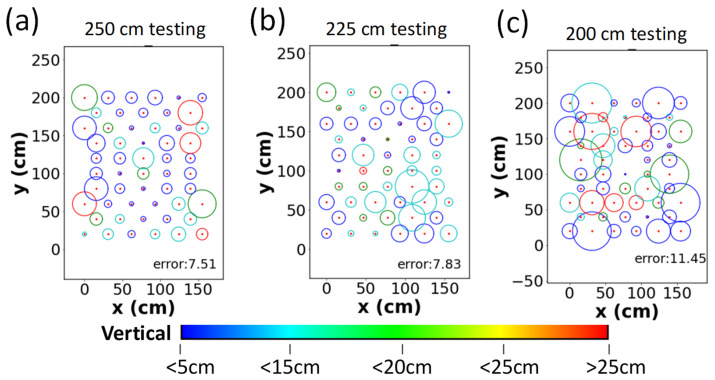
Experimental average error distributions of the testing points using the TSNN with RISE at the (**a**) 250 cm, (**b**) 225 cm, (**c**) 200 cm Rx planes.

**Figure 10:**
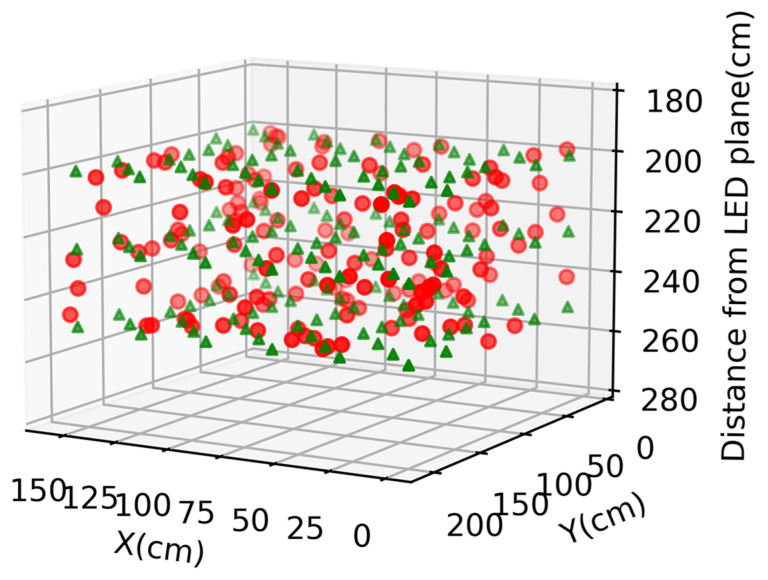
Three-dimensional visualization combining the 250, 225, and 200 cm Rx planes using the TSNN with RISE; green triangles and red circles are actual and predicted 3D locations.

When the TSNN model with the RISE is employed finally, the mean and standard deviation of errors for the testing data set in the vertical direction (*z*-direction) are the same as the TSNN model without the RISE since they are obtained before the RISE process. The mean and standard deviation of errors and for the horizontal direction (*xy*-direction) are 8.91 and 5.81 cm, respectively. Figure 9a–c show the average error distributions of the testing data set at different Rx planes. It is observed that when the RISE is used, the positioning error as well as the error variation can be significantly reduced. Figure 9a–c also illustrate the average error at each of the Rx planes, and the average errors are 7.51, 7.83, and 11.45 cm at the 250, 225, and 200 cm Rx planes, respectively. Comparing with the results using the one stage NN model, the error reductions are 37.1%, 40.6%, and 36.1% at the 250, 225, and 200 cm Rx planes when using the TSNN with RISE. Figure 10 is the 3D visualization combining the 250, 225, and 200 cm Rx planes using the TSNN with RISE, in which the green triangles and red circles are the actual and predicted 3D locations.

We also summarize the mean and the standard deviation of errors in Table 2. The results are obtained by averaging the values in all the Rx planes. We can observe that when using the TSNN with RISE, the mean positioning errors in both the training and the testing data set are significantly reduced. Comparing with the results using the one stage NN model, the mean errors in the training data set and testing data set are reduced by 67.0% and 22.7% when using the TSNN with RISE in the *z*-direction, and the mean errors in the training data set and testing data set are reduced by 46.9% and 30.3% in the *xy*-direction. It is also worthwhile to note that although the positioning errors in both vertical and horizontal directions are >8 cm, this seems to be much larger than the error >2 cm reported in the 3D VLIP systems [25,26]. However, the dimensions of the unit cell employed here is 2 × 1.5 × 3 m^3^, which is about 50 and 48 times larger than that in [25,26], respectively. We believe that the proposed work cannot only reduced the 3D positioning error and error fluctuations; but also can be employed in practical indoor environments.

**Figure 11:**
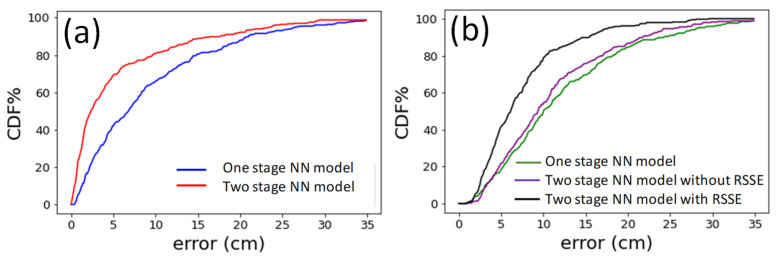
CDF of measured error at (**a**) *z*-direction (**b**) *xy*-direction using different NN models.

Figure 11a,b present the cumulative distribution function (CDF) of the measured positioning error in the *z*-direction and *xy*-direction, respectively, using different NN models. For *z*-direction, when using the one stage NN model, the positioning error of 90% of the experimental data is within 21 cm; while using the two stage NN model, the error is reduced to 17.6 cm. For *xy*-direction, when using the one stage NN model, the positioning error of 90% of the experimental data is within 24.2 cm; while using the TSNN model without and with the RISE, the errors are within 21.7 cm and 15.3 cm, respectively. Hence, at the CDF at 90% positioning error, the TSNN model with RISE can reduce the errors by 36.78% when compared with that in the one stage NN model.

It is believed that the noise can be reduced by increasing the signal-to-noise ratio (SNR) of the received optical signals, which can be increased by increasing the emitted optical powers from the LEDs; as well as reducing the thermal noise and shot noise of the Rx, as discussed in [27]. In a practical indoor environment, it may not be easy to increase the emitted optical powers from the LEDs. In this case, the field-of-view (FOV) of the LED could be reduced so that the light launched into the Rx can be more intense. The FOV of our LED is 64°. Although reducing the FOV of LED can increase the received SNR, it will increase the size of the light non-overlap zone as illustrated in Figure 3b,c. Hence, the proposed RISE is necessary to mitigate the light non-overlap zone.

**Figure 12:**
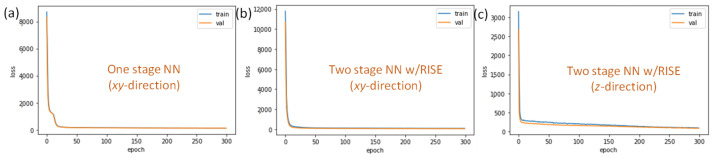
Loss performance during the training and validation processes using (**a**) one stage NN (*xy*), (**b**,**c**) TSNN with RISE (*xy* and *z*).

In the VLP experiments, our results show that the loss curves in all the cases (including one stage NN, TSNN without RISE and TSNN with RISE) converge stably within 50 epochs. Figure 12a–c illustrate some examples of the loss performance during the training and validation processes using the one stage NN (*xy*), TSNN with RISE (*xy* and *z*), respectively, showing that the loss curves converge stably very quickly. Here, the loss function and optimizer are mean squared error (MSE) and Adam optimizer respectively.

**Figure 13:**
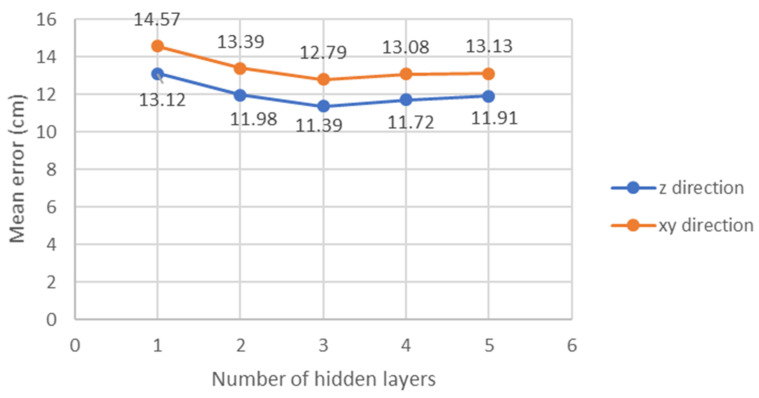
Mean error of the one stage NN using a different number of hidden layers.

As shown inside the VLP unit cell in Figure 1b, there are 58 training location points and 54 testing location points for each layer (i.e., Rx plane), and there are 3 layers in total. Each location point is measured by 20 times experimentally. Therefore, the training set has a total of 3480 data (58 locations × 20 measurements × 3 layers), and the testing set has 3240 data (54 locations × 20 measurements × 3 layers). We now describe our procedure to reduce the mean positioning error by optimizing the number of hidden layers and number of nodes (i.e., neurons) in each hidden layer. By optimizing these parameters, the complexity of the NN model can be reduced. We use the one stage NN as an example. We use multiples of 16 as the number of nodes in each hidden layer. First, we try a different number of hidden layers as shown in Figure 13. We can observe that using three hidden layers can achieve the lowest mean errors in both the *xy*- and *z*-directions. Then, we try several combinations of nodes for the three hidden layers, including (32, 16, 8), (64, 32, 16), (128, 64, 32) … etc. Additionally, by comparing the mean errors in these combinations, nodes (32, 16, 8) has the lowest mean error while using a small number of nodes. Here, a laptop computer with CPU (Intel^®^ i5-8265u), RAM of 20 GB, and Windows 10 home edition is utilized for the model training and validation. The training times for each model is within 50 s. After the NN model has been trained, the location prediction time is within 1 s.

It is also worthwhile to point out that the unit cell is not a perfect rectangle. Our idea is to study that high positioning accuracy can still be obtained, even though the LED arrangement is a non-perfect rectangle, which could be more realistic in a practical environment, since the machine learning model is built from the actual indoor environmental training data, and the RSS features extracted from the training data have already taken into account the non-perfect rectangular LED arrangement. High positioning accuracy using a TSNN model with RISE can be achieved.

## 4. Conclusions

We proposed and illustrated experimentally a 3D VLIP system utilizing TSNN model and RISE to alleviate the light non-overlap zones in VLIP system. The RISE can effectively alleviate the light non-overlap zones caused by the finite FOV of the LED lamps. Additionally, a one stage NN model and TSNN without the RISE were also comparing with the proposed scheme, and the results showed that the proposed scheme outperforms the others. The test-bed was a practical room with dimensions of 200 × 150 × 300 cm^3^. For the one stage NN model, although we only needed to run the NN model once, it was observed that the weights of the NN model have to be adjusted by considering the errors in three directions, so the average error in the *z*-direction and *xy*-direction were 11.39 and 12.79 cm, respectively. For the two stage NN model only, the average error in the *z*-direction and *xy*-direction were reduced to 8.80 and 12.56 cm, respectively. By employing the RISE to alleviate the light non-overlap region at different heights, the average error in the horizontal direction can be further reduced to 8.91 cm. Compared with the results using the one stage NN model, the mean errors in the training data set and testing data set were reduced by 67.0% and 22.7% when using the TSNN with RISE in the *z*-direction, and the mean errors in the training data set and testing data set were reduced by 46.9% and 30.3% in the *xy*-direction. Moreover, the CDF P90, the TSNN model with RISE, can reduce the errors by 36.78% when compared with that in the one stage NN model.

## Figures and Tables

**Table 1:** Recent 3D RSS VLIP systems.

Year	Scheme [Ref.]	2D/ 3D	Coverage (m^3^)	Accuracy	Light Non-Overlap Solution	Sim./ Exp’t
2017	RSS/CDMA [19]	3D	3 × 3 × 4	3.18 cm (horizontal); 7.06 cm (vertical)	---	Exp’t
2018	RSS/PASS [20]	3D	9 × 9 × 4	10 cm	---	Sim.
2019	RSS/Trilateration + NLLS [21]	3D	5 × 5 × 5	5.8 cm	---	Sim.
2019	RSS/Trilateration + NLLS [22]	3D	4 × 4 × 5	12.7 cm	---	Exp’t
2019	RSS/ANN + PDOA [23]	3D	4 × 4 × 2.5	12 cm	---	Sim.
2019	RSS/DNN [24]	3D	1.2 × 1.2 × 2	11.93 cm	---	Exp’t
2019	RSS/ANN [25]	3D	1/2 × 0.9 × 1 × 0.4	1 cm	---	Exp’t
2020	RSS/KRR [26]	3D	1/2 × 0.5 × 0.5 × 1.5	1.96 cm (horizontal); 2.16 cm (vertical)	---	Exp’t
This work	RSS/TSNN + RISE	3D	2 × 1.5 × 3	8.91 cm (horizontal); 8.8 cm (vertical)	Yes	Exp’t

**Table 2:** Mean error and standard deviation of different schemes.

Schemes	Directions	Train (Mean) (cm)	Test (Mean, Std.) (cm)
One stage NN	*z*-direction	9.02	11.39, 9.10
	*xy*-direction	11.48	12.79, 7.99
TSNN w/o RISE	*z*-direction	2.98	8.80, 9.90
	*xy*-direction	9.72	12.56, 8.30
TSNN w/ RISE	*z*-direction	2.98	8.80, 9.90
	*xy*-direction	6.1	8.91, 5.81

## Data Availability

The data presented in this study are available from the first author upon request.
